# Elevated levels of circulating betahydroxybutyrate in pituitary tumor patients may differentiate prolactinomas from other immunohistochemical subtypes

**DOI:** 10.1038/s41598-020-58244-8

**Published:** 2020-01-28

**Authors:** Omkar B. Ijare, Cole Holan, Jonathan Hebert, Martyn A. Sharpe, David S. Baskin, Kumar Pichumani

**Affiliations:** 1 Kenneth R. Peak Brain and Pituitary Tumor Treatment Center, Department of Neurosurgery, Houston Methodist Neurological Institute, Houston Methodist Hospital and Research Institute, Houston, TX USA; 2 Weill Cornell Medical College, New York, NY USA

**Keywords:** Physiology, Pituitary diseases

## Abstract

The diagnosis of various histological subtypes of pituitary tumors is made using serum based hormone panel test. However, certain subtypes secrete more than one hormone, making the diagnosis ambiguous. Here, we performed ^1^H-NMR based metabolomic analysis of serum and whole-blood from luteinizing/follicle-stimulating (LH/FSH)-secreting (n = 24), prolactinomas (n = 14), and non-functional (NF) (n = 9) tumors. We found elevated levels of betahydroxybutyrate (BHB) in serum and whole-blood (WB) of prolactinomas (0.481 ± 0.211/0.329 ± 0.228 mM in serum/WB), but it was statistically significant (p ≤ 0.0033, Bonferroni correction) only in serum when compared with LH/FSH-secreting tumor patients (0.269 ± 0.139/0.167 ± 0.113 mM in serum/WB). Phenylalanine in NF tumors was found to be elevated in both serum and WB when compared with prolactinomas but it met the statistical significance criteria (p ≤ 0.0028) only in the serum. Alanine (p ≤ 0.011), tyrosine (p ≤ 0.014) and formate (p ≤ 0.011) were also elevated in NF tumors but none showed statistically significance when compared with prolactinomas. Quantification of BHB and the above amino acids in the circulation may aid in the development of blood-based *in vitro* diagnostic methods which can supplement the currently used serum hormone panel in the diagnosis of various subtypes of pituitary tumors.

## Introduction

The pituitary gland secretes a range of hormones including growth (GH), adrenocorticotropic (ACTH), prolactin (PRL), luteinizing (LH), follicle-stimulating (FSH), and thyroid-stimulating (TSH) hormones^1^. PRL-secreting (prolactinomas) and LH/FSH-secreting are the most common subtypes of pituitary adenomas^2,3^. Non-functional (NF) pituitary adenomas are another common subtype of pituitary adenomas which do not secrete any hormones^4^. Although pituitary adenomas are generally benign tumors, they pose a major health challenge in patients through the oversecretion of hormones that may lead to infertility, sexual dysfunction, gigantism and other health issues such as vision problems, depression and osteoporosis^3,5^. Unlike other human diseases, there has not been any patient-derived pituitary tumor cell lines or mouse models currently available which limit the ability to understand molecular mechanisms behind tumor growth and also in discovering new diagnostic markers of pituitary adenomas. MRI is commonly used in the diagnosis of pituitary adenomas. However, it may be very difficult to detect microadenomas in the MRI scan^3^. I*n vivo* ^1^H magnetic resonance spectroscopy (MRS) will be of some help in diagnosing hemorrhagic pituitary macroadenomas^6^. Currently, the clinical MRI scanners use lower magnetic field strength which makes it difficult to fully characterize pituitary adenomas using *in vivo* ^1^H MRS. Whereas, *ex vivo* high resolution MRS makes use of higher magnetic field strength which provides higher sensitivity and better resolution resulting in superior characterization of pituitary tumors^2^. The metabolite information obtained from *ex vivo* ^1^H MRS on pituitary adenomas will further aid in the definitive characterization of pituitary tumors using *in vivo* ^1^H MRS. Recently, we have identified a set of metabolites using^1^H MRS in surgically resected tumor tissues that distinguish various immunohistochemical (IHC) subtypes of pituitary tumors^2^. However, very limited accessibility of the pituitary gland complicates the use of tissue biopsies for diagnostics. Currently, diagnosis of pituitary tumors is based on the abnormal serum hormone panel. Certain subtypes of pituitary tumors oversecrete more than one hormone and NF pituitary adenomas do not secrete any hormones, making the hormone panel based diagnostic methods ambiguous. Blood metabolomics has been used to identify cancer biomarkers and in the case of pituitary tumors it can be of great value by supplementing to the current clinical diagnostics^7^. In addition, whole blood (WB) contains red blood cells (RBCs) and other cellular components of blood such as white blood cells and platelets which can further add to the information derived from serum metabolomics^8,9^. Also, RBCs are known to contain high concentrations of cofactors involved in redox reactions and cellular energy metabolism. Redox mechanisms are fundamental biochemical reactions that are essential to all living cells and directly involved in the homeostasis of key metabolites such as pyruvate and lactate which play pivotal role in various metabolic pathways^10^. Here, we studied the metabolome of serum and WB obtained from patients with prolactinomas, LH/FSH-secreting and NF pituitary adenomas using ^1^H NMR spectroscopy with the aim of quantitative identification of metabolite markers that can be of potential value in the development of *in vitro* diagnostics to differentiate various pituitary tumor subtypes.

## Materials and Methods

### Patients and blood samples collection

Blood samples were collected from 47 patients (prolactinoma = 14; LH/FSH-secreting = 24; and NF = 9) undergoing transsphenoidal selective adenomectomy for the surgical excision of pituitary adenomas at the Houston Methodist Hospital. Informed consent was obtained from each patient following an Institutional Review Board protocol approved by the Houston Methodist Hospital and Research Institute. The work described in this article has been carried out in accordance with “The Code of Ethics of the World Medical Association (Declaration of Helsinki) for experiments involving humans”. The diagnosis of pituitary tumors was made on the basis of clinical, biochemical, imaging and IHC criteria. The patient characteristics along with tumor type (IHC), MIB-1 index, PRL and betahydroxybutyrate (BHB) levels are provided in Table 1. Prolactinoma was predominantly observed in females, and 11 out of 14 prolactinoma patients enrolled in this study were females.

**Table 1:** Characteristics of PRL and LH/FSH secreting tumor patients showing their sex, age, tumor type, MIB-1 index (%) along with BHB and PRL levels in the serum and whole blood (WB).

Patient #	Sex	Age (years)	Tumor type (IHC)	MIB-1 (%)	BHB* (mM/L)		PRL (ng/mL)	Medical Therapy#
					Serum	WB		
1	M	45	LH	1–2	0.058	0.042	8.0	No
2	F	26	LH	2	0.106	0.054	**37.0**	No
3	F	77	LH^Ψ^	1	**0.438**	**0.255**	**30.0**	No
4	F	64	FSH^Ψ^	1	**0.482**	**0.418**	**37.0**	No
5	M	61	FSH	1	0.373	0.072	Normal	No
6	M	46	FSH	2–3	0.261	0.158	14.1	No
7	F	57	FSH	1	0.455	0.287	NA	No
8	F	77	FSH^Ψ^	1–2	**0.484**	**0.287**	**37.0**	No
9	F	66	LH/FSH	2–3	0.331	0.225	Normal	No
10	M	81	LH/FSH	1	0.215	0.188	NA	No
11	F	25	LH/FSH	2	0.223	0.166	**148.0**	No
12	F	54	LH/FSH	1	0.135	0.086	**51.2**	No
13	M	53	LH/FSH	2	0.155	0.134	23.3	No
14	M	61	LH/FSH	1–2	0.304	0.252	NA	No
15	M	47	LH/FSH	1	0.268	0.103	<1	No
16	F	65	LH/FSH	1	0.455	0.291	NA	No
17	M	59	LH/FSH	1–2	0.160	0.054	6.0	No
18	M	55	LH/FSH	2	0.104	0.037	23.0	No
19	M	79	LH/FSH	1	0.282	0.161	20.4	No
20	M	72	LH/FSH	1	0.091	0.038	NA	No
21	F	65	LH/FSH	1	0.560	0.334	NA	No
22	M	62	LH/FSH	1	0.210	0.092	17.4	No
23	M	71	LH/FSH	1–2	0.215	0.085	**39.4**	No
24	M	65	LH/FSH	2	0.238	0.105	13.5	No
25	F	49	PRL	3–4	0.930	0.937	**79.4**	No
26	F	34	PRL	2	0.656	0.482	**274**	Yes
27	F	36	PRL	3	0.640	0.416	**197.2**	No
28	F	22	PRL	NA	0.232	0.122	Elevated	Yes
29	F	33	PRL	No staining	0.624	0.444	**114**	Yes
30	F	50	PRL	1	0.614	0.513	NA	No
31	F	37	PRL	1–2	0.549	0.380	**82.5**	No
32	M	22	PRL	5–7	0.179	0.133	**115.3**	Yes
33	M	59	PRL	No staining	0.429	0.175	**249.0**	Yes
34	F	45	PRL	2	0.268	0.104	**41**	No
35	F	33	PRL	1	0.291	0.209	**82.2**	Yes
36	F	27	PRL	1	0.294	0.120	**64.0**	Yes
37	M	24	PRL	5–7	0.463	0.216	**1029.0**	Yes
38	F	53	PRL	1	0.567	0.355	10.0	No
39	F	46	NF	1	0.169	0.159	9.0	No
40	M	37	NF	3	0.078	0.057	7.6	No
41	F	39	NF	1	0.058	0.0416	NA	No
42	M	42	NF	1	0.470	0.313	7.4	No
43	M	65	NF	2	0.188	0.153	NA	No
44	F	75	NF	1	0.669	0.459	NA	No
45	F	72	NF	2	0.439	0.213	NA	No
46	F	73	NF	1	0.318	0.164	NA	No
47	M	73	NF	1	0.849	No sample	8.0	No

(IHC, Immunohistochemistry; LH, luteinizing hormone; FSH, follicle stimulating hormone; PRL, prolactin; NA, not available; PRL-reference range = 2–23 ng/mL; *, BHB levels measured by ^1^H NMR spectroscopy; Ψ, IHC was positive for LH/FSH, also showed scattered immunoreactivity for PRL, and these patient also showed elevated levels of PRL and BHB similar to prolactinoma patients; #, patients were treated with Cabergoline, 2–4 mg/week).

Two vials of blood (4.0 mL each) were collected in serum separator tubes (BD Diagnostics, NJ) during surgical resection of pituitary tumors. One of the vials was immediately snap-frozen, while the other vial was kept at room temperature for 20–30 min., and then the serum was separated by centrifugation (RCF = 1,800 g for 15 minutes at 4 °C), transferred to cryovials and all specimens were stored at −80 °C until further analysis.

### Chemicals

Methanol, chloroform, chloroform-*d* (CDCl_3_), Na_2_HPO_4,_ and NaH_2_PO_4_ were purchased from Millipore Sigma (St. Louis, MO, USA). D_2_O, DCl, and NaOD were purchased from Cambridge isotope laboratories (Tewksbury, MA, USA). 3-(trimethylsilyl)-1-propane sulfonic acid-d_6_ sodium salt (DSS-*d*_6_) solution was purchased from Chenomx Inc. (Edmonton, Canada).

### Preparation of phosphate buffer solution

Phosphate buffer solution (100 mM) was prepared by dissolving 2.24 g of anhydrous Na_2_HPO_4_ and 0.5 g of anhydrous NaH_2_PO_4_ in 200 g of D_2_O. A 4.99 mM solution of DSS-*d*_6_ was added to the buffer solution to obtain a final DSS-*d*_6_ concentration of 0.1 mM. The pH of the resulting phosphate buffer was 7.40.

### Methanol-chloroform extraction

To 0.25 ml of the serum or WB, 0.5 mL of methanol and 0.5 mL chloroform (both solvents kept at 4 °C) were added in the ratio 1:2:2 (sample:methanol:chloroform, v/v/v)^8^. The sample-solvent mixtures were vortexed for 1 minute and kept on ice for 2 minutes. This step was repeated twice and the resultant mixtures were incubated at −20 °C for 20 minutes followed by centrifugation at 10,000 g (RCF) for 15 minutes. Only the upper aqueous methanolic layers were carefully collected and the solvents were dried in a CentriVap^®^ vacuum concentrator (Labconco Corporation, Kansas City, MO). The dried residue from each sample collected from the above step (for each sample) was reconstituted in 600 µL phosphate buffer (pH = 7.40) containing 0.1 mM DSS-*d*_6_ (internal standard).

### ^1^H NMR experiments

One-dimensional (1D) ^1^H NMR experiments of serum and WB extracts were performed on a Bruker 600 MHz spectrometer (^1^H Larmor frequency) equipped with a cryogenically-cooled ^1^H/^13^C detection probe (Bruker Biospin, Billerica, MA) at 300 K. The sample solutions prepared above were taken in 5 mm NMR tubes and the ^1^H NMR spectra were obtained by using 1D NOESY (nuclear Overhauser spectroscopy) and CPMG (Carr-Purcell-Meiboom-Gill) pulse sequences with water suppression by presaturation. Both NOESY and CPMG experiments were performed using 32,768 time domain data points, with a 9615.38 Hz spectral width. A 90° excitation pulse with a pulse-width of 10 µs and an inter-pulse delay of 5 s were used. In 1D NOESY experiments, a mixing time of 100 ms was used while in CPMG experiments a total echo-time of 160 ms was used to attenuate broad signal arising from residual lipid components in the serum extracts. The data were zero-filled (2× time domain points), an exponential window function with line broadening of 0.3 Hz was applied, and Fourier transformed. DSS-*d*_6_ (resonating at 0 ppm) was used as an internal chemical shift and concentration reference (0.1 mM). Peak areas of ^1^H signals were measured by using Bruker TopSpin version 3.5 software program.

### Quantification of aqueous metabolites

We have identified water-soluble metabolites (in the aqueous methanol layers of the serum and WB extracts) using ^1^H chemical shifts data of pituitary tumors from our recent study and other reported data^2,8,10,11^. Quantification of metabolites was performed as described in our recent publication^2^. Details of quantified metabolites, and their corresponding ^1^H NMR chemical shifts (of peaks used for quantification) are given in Supplementary Tables S1 and S2.

### Statistical analysis

All statistical analyses were performed using Microsoft Excel software (Microsoft Office 2013). The means and standard deviations of metabolites quantified were compared between LH/FSH-secreting tumors, prolactinomas, and NF tumors. The Bonferroni correction (p-value ≤ 0.0038) was applied to the p-values obtained from Student’s t-test to determine the significance level by taking multiple metabolite testing into consideration (Supplementary Tables S1 and S2)^12^. The diagnostic accuracy of BHB in the differentiation of prolactinomas from other pituitary adenomas was assessed using receiver operative characteristic (ROC) analysis^13,14^. The area under curve (AUC) was computed to determine the diagnostic accuracy of the test. ROC curves were generated by plotting the true positive rate and the false positive rate.

## Results and Discussion

**Figure 1:**
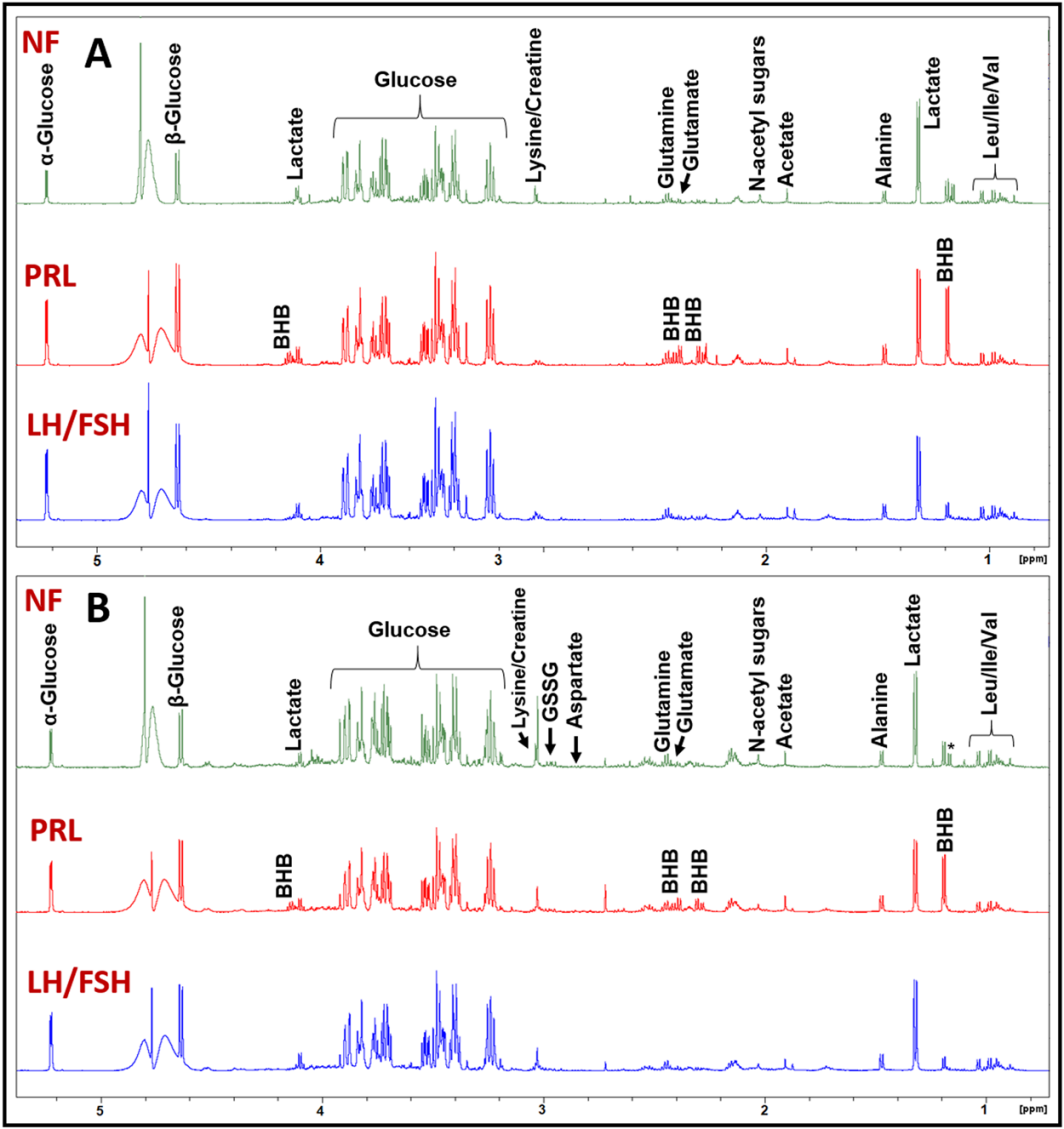
Representative ^1^H NMR spectra of aqueous methanol extracts of (**A**) serum and (**B**) whole blood (WB) showing spectral profiles of LH/FSH-secreting pituitary tumor, prolactinoma (PRL-secreting), and Non-functional (NF) pituitary tumor patients. The BHB is highly elevated in the serum and WB of prolactinoma patient. *Refers to solvent impurity signal.

**Figure 2:**
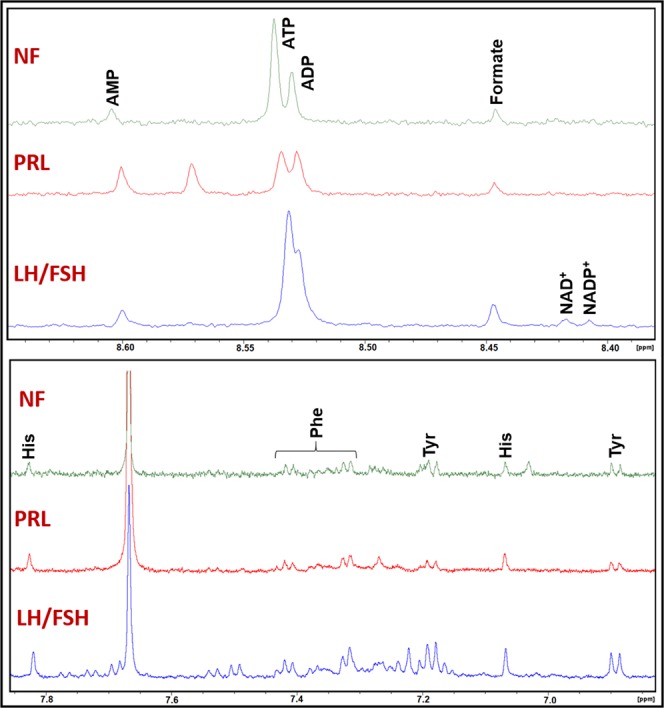
Representative ^1^H NMR spectra of aqueous methanol extracts of whole blood (WB) showing signals arising from cofactors ATP, ADP, AMP, NAD^+^, and NADP^+^ in LH/FSH-secreting pituitary tumor, prolactinoma, and NF pituitary tumor patients. Also shown are the ^1^H NMR signal assignments for aromatic amino acids tyrosine, phenylalanine, and histidine.

**Figure 3:**
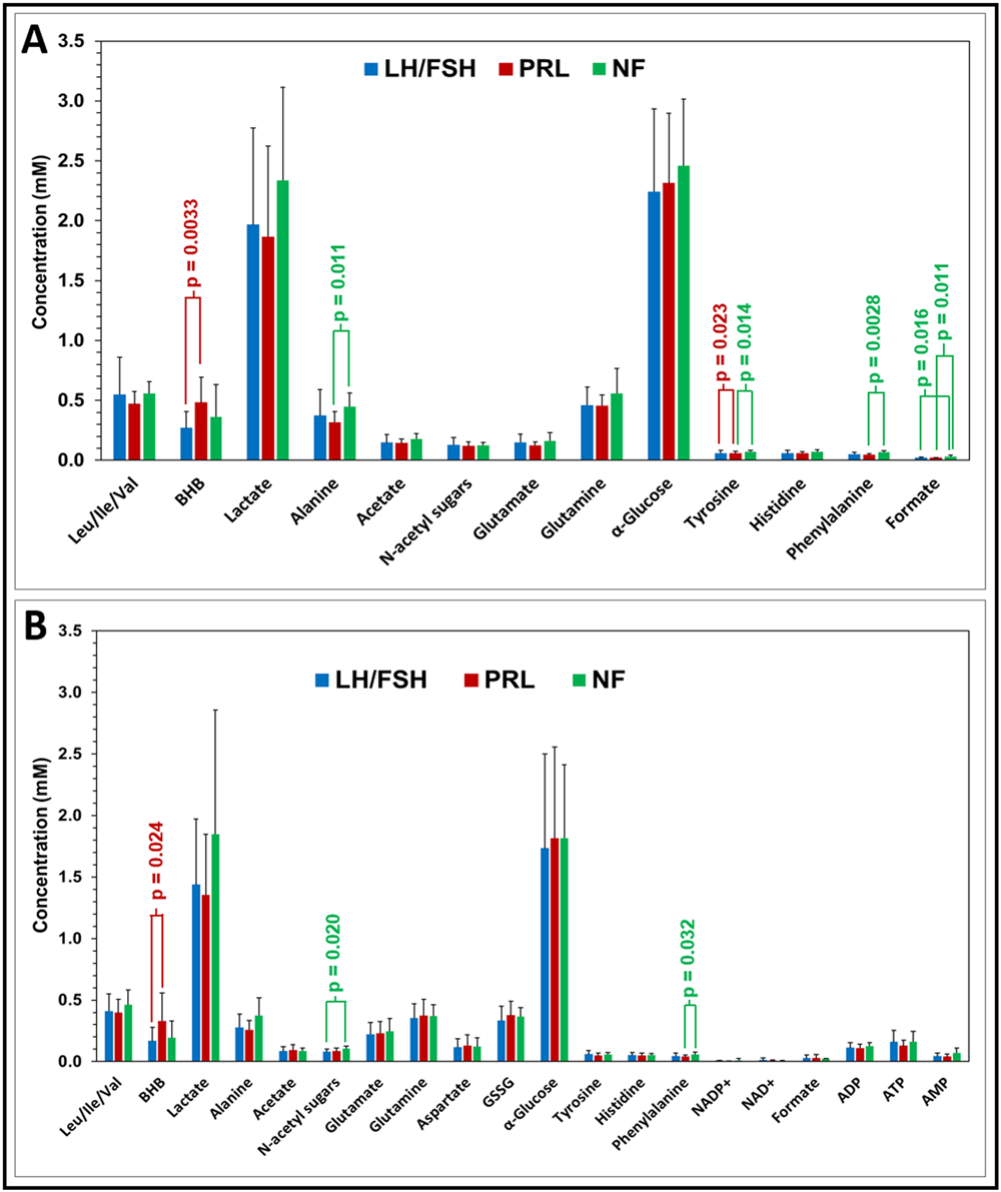
Charts showing the concentrations of various water soluble metabolites quantified in (**A**) serum and (**B**) WB of LH/FSH-secreting pituitary tumor, prolactinoma, and NF pituitary tumor patients. BHB was significantly elevated in both serum and WB of prolactinoma patients compared to LH/FSH-secreting pituitary tumors. Total glucose concentration can be determined from α-Glucose (36% anomeric contribution) using the relation, [Total Glucose] = [α-Glucose] × (100/36).

Figure 1A shows the ^1^H NMR spectral profile of metabolites in the aqueous-methanolic extract of serum from LH/FSH-secreting, prolactinoma, and NF pituitary tumor patients. Similarly, Fig. 1B shows the ^1^H NMR spectral profile of an aqueous methanolic extract of WB from the same patients. Both serum and WB showed the presence of leucine (Leu), isoleucine (Ile), valine (Val), BHB, lactate, alanine, acetate, N-acetyl sugars, glutamate, glutamine, lysine/creatine, glucose, tyrosine, histidine, phenylalanine, and formate. In addition to the above metabolites, the WB showed the presence of peaks arising from aspartate, glutathione (GSSG, oxidized form) and cofactors involved in the cellular redox reactions – ATP, ADP, AMP, NAD^+^, and NADP^+^. The NMR spectral assignments of aromatic amino acids (tyrosine, phenylalanine, and histidine) and cofactors (ATP, ADP, AMP, NAD^+^, and NADP^+^) are shown in Fig. 2 for WB extracts of LH/FSH-secreting, prolactinoma, and NF pituitary tumor patients. The mean metabolite concentrations in serum and WB of LH/FSH-secreting, prolactinoma, and NF tumor patients are shown in Fig. 3A,B respectively (also see Supplementary Tables S1 and S2). From Fig. 3A,B, we can see that BHB was significantly elevated only in serum of prolactinoma patients compared to the LH/FSH-secreting patients (p = 0.0033). Although in NF tumors, tyrosine (p ≤ 0.023) and formate (p ≤ 0.011) were elevated in serum, and N-acetyl sugars were found to be elevated in WB, none of them met the statistical significance criteria (p ≤ 0.0038) used in this study to account for multiple metabolite testing while comparing with LH/FSH-secreting tumors. On the other hand, phenylalanine in NF tumors was found to be elevated in both serum and WB when compared with prolactinomas but it met the statistical significance criteria (p ≤ 0.0028) only in the serum. Also, alanine (p ≤ 0.011), tyrosine (p ≤ 0.014) and formate (p ≤ 0.011) showed statistically nonsignificant elevated levels in the serum of NF tumors when comparing with prolactinomas.

In WB extracts, the levels of ATP and ADP in three types of tumor patients were comparable, but the levels of AMP were slightly elevated in NF tumors (Table S2). The ATP/ADP ratio was lower in prolactinoma patients compared to LH/FSH-secreting and NF tumor patients (Fig. 3B; Table S2). ATP/ADP ratio plays a key role in the cellular metabolism that determines the balance in the relative fluxes between glycolysis and oxidative phosphorylation^15^. In this study, we observed a slight decrease in the ATP/ADP ratio and the lactate levels in prolactinoma patients along with slight increase in the aspartate levels when compared with LH/FSH-secreting tumors. This suggests that prolactinomas may have slightly downregulated glycolysis than the mitochondrial oxidative metabolism. On the other hand, NF tumors showed elevated levels of lactate when compared with both prolactinomas and LH/FSH-secreting tumors, indicating an upregulated Warburg glycolysis. Moreover, we also observed slightly elevated levels of NADP^+^ in LH/FSH secreting tumors compared to prolactinomas and NF tumor patients, but NAD^+^ levels were similar in all three types of tumors (Fig. 3B, Table S2). Together, this may be indicative of enhanced glycolysis in NF pituitary tumors than other subtypes. Pituitary tumors are often small in size and procuring enough tumor tissues for *ex vivo* metabolomic analysis will be challenging. As described in this study, detection of cofactors and antioxidants in WB using a simple ^1^H NMR based method may be a valuable alternative approach towards understanding their role in various IHC subtypes of pituitary tumors.

Glucose is the primary energy source for the central nervous system. It is known that under prolonged fasting brain switches to the utilization of ketone body (BHB) as an energy substrate which directly enters the citric acid cycle in the mitochondria. It was assumed that brain tumors lacked the ability to efficiently metabolize ketone body due to dysfunctional mitochondria. Our recent surprising findings demonstrate that malignant brain tumors are fully capable of oxidizing BHB, glutamine and acetate as alternate fuels^16,17^. In a similar fashion, we hypothesize that elevated levels of circulating BHB in prolactinoma patients may be taken up preferentially and utilized by the tumors to meet their increased bioenergetic requirements.

The BHB levels in fasting individuals generally do not exceed 0.2 mM^18^. In the current study, we observed that the levels of BHB were elevated in prolactinoma patients (0.481 ± 0.211 mM and 0.329 ± 0.228 mM in serum and WB respectively) compared to the LH/FSH-secreting tumor patients (0.269 ± 0.139 mM and 0.167 ± 0.113 mM in serum and WB respectively) and NF tumors (0.360 ± 0.272 mM and 0.195 ± 0.137 mM in serum and WB respectively). The difference in the levels of BHB in LH/FSH-secreting and prolactinomas was statistically significant only in serum (p = 0.0033) with Bonferroni correction (corrected p ≤ 0.0038). We did not see a statistically significant difference between BHB levels in NF tumors when compared with LH/FSH-secreting tumors or prolactinomas. BHB is synthesized in the liver via the oxidation of fatty acids during starvation or under the conditions of depleted dietary carbohydrates/glycogen in the body^19^ and is transported to the brain as a fuel. It is also synthesized endogenously in astrocytes and is supplied to other brain cells^20^. A recent *in vitro* study carried out in dairy cow anterior pituitary (DCAP) cells has shown a link between BHB and PRL secretion^21^. The authors have shown that BHB can inhibit PRL gene transcription and secretion via cAMP/protein kinase signalling pathway^21^. Secretion of PRL was decreased in DCAP cells in a dose dependent manner when treated with BHB. On the contrary, in this study prolactinoma patients showed elevated levels of both BHB and PRL. Although the levels of both PRL and BHB in prolactinoma patients were high, we could not find any correlation between the levels of PRL and BHB. This could be due to the fact that most of prolactinoma patients were on cabergoline therapy (Table 1) that is used to lower PRL levels. We also observed that 7 patients with LH or FSH secreting tumors also showed elevated levels of PRL (patients #2, 3, 4, 8, 11, 12 and 23; Table 1). It is interesting to note that the BHB levels in 4 of these 7 patients (#2, 11, 12 and 23), were in the normal fasting range^18^. However, in the remaining 3 patients (#3, 4, and 8), BHB levels were found to be elevated. These 3 patients also showed scattered positive immunoreactivity for PRL which may be the reason for the elevation in the BHB levels .

**Figure 4:**
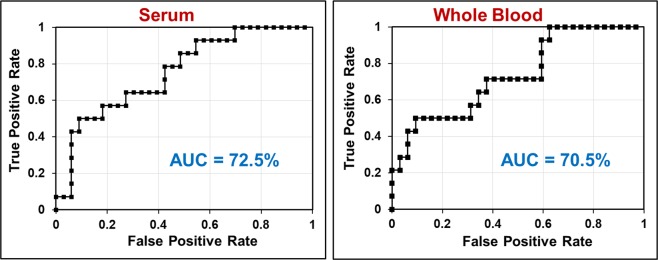
ROC curves for BHB levels in serum and whole blood (WB) showing diagnostic potential of BHB in differentiating prolactinomas from LH/FSH-secreting and NF pituitary adenomas.

To assess the diagnostic accuracy of BHB in differentiating prolactinomas from LH/FSH-secreting and NF tumors, we performed ROC analysis and generated the ROC curves. Figure 4 shows the ROC plots of BHB levels determined from serum and WB of tumor patients. The area under the curve (AUC) for serum and WB were 72.5% and 70.5% respectively, indicating that the BHB can be a good metabolic marker for the differential diagnosis of prolactinomas from other subtypes of pituitary adenomas. The observation of elevated circulating BHB levels may imply a possible association with PRL secretion in pituitary tumor patients. The reason for the elevated levels of circulating BHB observed only in prolactinoma patients (Table 1) is not fully understood. Further studies are needed to unravel the relationship between BHB and PRL levels in the circulation of prolactinoma patients. It could be possible that prolactinomas may preferentially utilize BHB as an alternate fuel for their growth similar to the malignant brain tumors^16,17^. Currently, prolactinomas are the only pituitary tumors which can be treated by medical therapy. Cabergoline and bromocriptine are the two drugs prescribed to the patients with PRL-secreting pituitary tumors. Our current observation of elevated levels of circulating BHB along with increased PRL secretion in prolactinoma patients suggest that modulating the levels of BHB in the circulation may have a therapeutic role in treating these patients.

## Conclusions

Elevated levels of BHB in the circulation distinguish prolactinomas from LH/FSH-secreting and NF tumors. Quantification of BHB levels in serum/WB may aid in the development of blood based non-invasive diagnostic methods which can augment the currently available serum hormone panel in the unambiguous diagnosis of PRL-secreting tumors. Although exact role of elevated BHB in prolactinoma patients is not fully clear, we hypothesize that higher levels of circulating BHB in prolactinoma patients may be taken up by the tumors to meet their increased energy requirements. In the serum of NF tumors the levels of alanine, phenylalanine and tyrosine were found to be elevated but only phenylalanine showed statistically significant elevation when compared with prolactinomas. Given a small sample size, we were unable to determine a definitive decision threshold for BHB in differentiating the prolactinomas from other pituitary tumor subtypes. However, we obtained an accuracy of ~72% using ROC analysis. We are continuing to enroll more prolactinoma, LH/FSH-secreting and NF pituitary tumor patients to further validate our preliminary findings. Also, we are recruiting other rarely occurring histological subtypes (GH-secreting and ACTH-secreting tumors) to test whether BHB or any other metabolites in the circulation play any physiological role in the diagnostics of these pituitary tumors.

## Supplementary information

Supplementary Dataset 1.

## Data Availability

^1^H NMR spectroscopic data from prolactinomas, LH/FSH secreting, and NF pituitary tumors generated for this are available in this article and its supporting information file.
