# Supplementary material for: Elevated levels of circulating betahydroxybutyrate in pituitary tumor patients may differentiate prolactinomas from other immunohistochemical subtypes
Source: Sci Rep. 2020 Jan 28;10:1334. doi: 10.1038/s41598-020-58244-8 (PMC6987215; doi:10.1038/s41598-020-58244-8)
Supplement: Supplementary file 1 — Supplementary Dataset 1. [file 41598_2020_58244_MOESM1_ESM.docx]

**Elevated levels of circulating betahydroxybutyrate in pituitary tumor patients may differentiate prolactinomas from other immunohistochemical subtypes**

Omkar B. Ijare^1^, Cole Holan^1^, Jonathan Hebert^1^, Martyn A. Sharpe^1^, David S. Baskin^1,2^, and Kumar Pichumani^1,2*^

^1^Kenneth R. Peak Brain and Pituitary Tumor Treatment Center, Department of Neurosurgery, Houston Methodist Neurological Institute, Houston Methodist Hospital and Research Institute, Houston, TX, USA, ^2^Weill Cornell Medical College, New York, NY, USA.

*Correspondence to:

Kumar Pichumani, Ph.D.

Kenneth R. Peak Brain and Pituitary Tumor Treatment Center

Department of Neurosurgery

Houston Methodist Research Institute and Hospital

6560 Fannin Street

Houston, TX 77030

Tel: 713 441 7190

Fax: (713) 793 7022

E-Mail: [kpichumani@houstonmethodist.org](mailto:kpichumani@houstonmethodist.org)

| **Metabolite (µmol/mL, serum)**  **(HMDB ID)** | **Chemical shift**  **(ppm)** | **LH/FSH-secreting tumor** | | **PRL-secreting tumor** | | **Non-functional (NF) tumor** | | **P-value** | | |
| --- | --- | --- | --- | --- | --- | --- | --- | --- | --- | --- |
|  |  | Mean | S.D. | Mean | S.D. | Mean | S.D. | LH/FSH  vs. PRL | LH/FSH  vs. NF | PRL  vs. NF |
| Leu/Ile/Val  (HMDB0013773/HMDB0000172/  HMDB0000883) | 0.887 | 0.549 | 0.312 | 0.469 | 0.104 | 0.556 | 0.098 | 0.2621 | 0.9219 | 0.0597 |
| Betahydroxybutyrate (BHB)  (HMDB0000357) | 1.189 | 0.269 | 0.139 | 0.481 | 0.211 | 0.360 | 0.272 | **0.0033** | 0.3635 | 0.2760 |
| Lactate ( HMDB0000190) | 1.319 | 1.965 | 0.811 | 1.865 | 0.756 | 2.336 | 0.778 | 0.7054 | 0.2462 | 0.1702 |
| Alanine (HMDB0000056) | 1.474 | 0.371 | 0.218 | 0.316 | 0.089 | 0.447 | 0.114 | 0.2826 | 0.2096 | **0.0111** |
| Acetate (HMDB0000042) | 1.907 | 0.146 | 0.069 | 0.141 | 0.037 | 0.178 | 0.043 | 0.7820 | 0.1289 | 0.0540 |
| N-acetyl sugars* ((HMDB0000215) | 2.031 | 0.128 | 0.063 | 0.117 | 0.036 | 0.126 | 0.022 | 0.4849 | 0.9174 | 0.4296 |
| Glutamate (HMDB0060475) | 2.338 | 0.148 | 0.072 | 0.123 | 0.032 | 0.161 | 0.069 | 0.1584 | 0.6359 | 0.1532 |
| Glutamine (HMDB0003423) | 2.455 | 0.458 | 0.151 | 0.454 | 0.091 | 0.556 | 0.210 | 0.9161 | 0.2260 | 0.1982 |
| α-Glucose^#^ (HMDB0000122) | 5.225 | 2.241 | 0.692 | 2.314 | 0.581 | 2.460 | 0.557 | 0.7294 | 0.3601 | 0.5547 |
| Tyrosine (HMDB0003831) | 6.869 | 0.057 | 0.027 | 0.056 | 0.020 | 0.073 | 0.011 | 0.8531 | **0.0227** | **0.0140** |
| Histidine (HMDB0000177) | 7.059 | 0.057 | 0.027 | 0.057 | 0.016 | 0.071 | 0.017 | 0.9527 | 0.0806 | 0.0653 |
| Phenylalanine (HMDB0000159) | 7.423 | 0.050 | 0.015 | 0.047 | 0.009 | 0.066 | 0.014 | 0.3375 | **0.0130** | **0.0028** |
| Formate (HMDB0000142) | 8.444 | 0.018 | 0.009 | 0.018 | 0.006 | 0.032 | 0.012 | 0.9735 | **0.0113** | **0.0114** |

**Table S1: Comparison of metabolite concentrations in serum samples of LH/FSH-secreting, prolactinomas (PRL-secreting), and non-functional (NF) tumors determined by ^1^H NMR spectroscopy. P-values are determined by Student’s t-test.**

*, N-acetyl methyl group of mobile N-acetyl glucosamine sugar residues on the glycan portions of glycoproteins (Otvos et al. 2015). ^#^, Total glucose can be determined from α-Glucose (36% anomeric contribution) using the relation, [Total Glucose] = [α-Glucose]×(100/36).

| **Metabolite (µmol/mL, serum)**  **(HMDB ID)** | **Chemical shift (ppm)** | **LH/FSH-secreting tumor** | | **PRL-secreting tumor** | | **Non-functional (NF) tumor** | | **P-value** | | |
| --- | --- | --- | --- | --- | --- | --- | --- | --- | --- | --- |
|  |  | Mean | S.D. | Mean | S.D. | Mean | S.D. | LH/FSH  vs. PRL | LH/FSH  vs. NF | PRL  vs. NF |
| Leu/Ile/Val  (HMDB0013773/HMDB0000172/  HMDB0000883) | 0.886 | 0.409 | 0.143 | 0.394 | 0.113 | 0.463 | 0.119 | 0.7342 | 0.3086 | 0.2072 |
| Betahydroxybutyrate (BHB)  (HMDB0000357) | 1.189 | 0.167 | 0.113 | 0.329 | 0.228 | 0.195 | 0.137 | **0.0236** | 0.6100 | 0.1001 |
| Lactate ( HMDB0000190) | 1.312 | 1.435 | 0.535 | 1.353 | 0.494 | 1.848 | 1.008 | 0.6362 | 0.2985 | 0.2251 |
| Alanine (HMDB0000056) | 1.473 | 0.278 | 0.109 | 0.258 | 0.075 | 0.374 | 0.142 | 0.5121 | 0.1109 | 0.0597 |
| Acetate (HMDB0000042) | 1.904 | 0.083 | 0.039 | 0.092 | 0.044 | 0.087 | 0.023 | 0.5167 | 0.7244 | 0.7174 |
| N-acetyl sugars* (HMDB0000215) | 2.029 | 0.079 | 0.024 | 0.083 | 0.028 | 0.104 | 0.023 | 0.6750 | **0.0203** | 0.0683 |
| Glutamate (HMDB0060475) | 2.340 | 0.220 | 0.098 | 0.228 | 0.097 | 0.246 | 0.105 | 0.8095 | 0.5539 | 0.7018 |
| Glutamine (HMDB0003423) | 2.446 | 0.352 | 0.119 | 0.370 | 0.136 | 0.371 | 0.090 | 0.6751 | 0.6426 | 0.9947 |
| Aspartate (HMDB0006483) | 2.795 | 0.115 | 0.069 | 0.128 | 0.088 | 0.120 | 0.073 | 0.6408 | 0.8591 | 0.8275 |
| GSSG (HMDB0000125) | 2.967 | 0.334 | 0.115 | 0.376 | 0.115 | 0.368 | 0.070 | 0.2878 | 0.3329 | 0.8379 |
| α-Glucose^#^ (HMDB0000122) | 5.226 | 1.733 | 0.766 | 1.813 | 0.742 | 1.815 | 0.596 | 0.7518 | 0.7592 | 0.9966 |
| Tyrosine (HMDB0003831) | 6.894 | 0.058 | 0.032 | 0.047 | 0.022 | 0.056 | 0.017 | 0.2439 | 0.8336 | 0.3180 |
| Histidine (HMDB0000177) | 7.064 | 0.051 | 0.024 | 0.047 | 0.025 | 0.052 | 0.015 | 0.5883 | 0.9072 | 0.5382 |
| Phenylalanine (HMDB0000159) | 7.422 | 0.044 | 0.024 | 0.039 | 0.014 | 0.058 | 0.019 | 0.4074 | 0.1185 | **0.0322** |
| Formate (HMDB0000142) | 8.448 | 0.006 | 0.005 | 0.005 | 0.003 | 0.011 | 0.016 | 0.2643 | 0.3985 | 0.2914 |
| NADP+ (HMDB0000217) | 8.407 | 0.012 | 0.019 | 0.007 | 0.007 | 0.003 | 0.004 | 0.2980 | 0.0637 | 0.1577 |
| NAD+ (HMDB0000902) | 8.418 | 0.026 | 0.027 | 0.026 | 0.032 | 0.020 | 0.005 | 0.9878 | 0.2971 | 0.4797 |
| ADP (HMDB0001341) | 8.516 | 0.110 | 0.042 | 0.109 | 0.032 | 0.125 | 0.029 | 0.9333 | 0.2906 | 0.2603 |
| ATP (HMDB0000538) | 8.541 | 0.159 | 0.095 | 0.127 | 0.046 | 0.161 | 0.083 | 0.1854 | 0.9355 | 0.3129 |
| AMP (HMDB0000045) | 8.598 | 0.043 | 0.026 | 0.039 | 0.023 | 0.070 | 0.040 | 0.6474 | 0.1023 | 0.0717 |

**Table S2: Comparison of metabolite concentrations in whole blood (WB) samples of LH/FSH-secreting, prolactinomas (PRL-secreting), and non-functional (NF) tumors determined by ^1^H NMR spectroscopy. P-values are determined by Student’s t-test.**

*, N-acetyl methyl group of mobile N-acetyl glucosamine sugar residues on the glycan portions of glycoproteins (Otvos et al. 2015). ^#^, Total glucose can be determined from α-Glucose (36% anomeric contribution) using the relation, [Total Glucose] = [α-Glucose]×(100/36).
